# TRANSHIATAL ESOPHAGECTOMY IN SQUAMOUS CELL CARCINOMA OF THE ESOPHAGUS: WHAT ARE THE BEST INDICATIONS?

**DOI:** 10.1590/0102-672020200004e1567

**Published:** 2021-03-22

**Authors:** Felipe Monge VIEIRA, Marcio Fernandes CHEDID, Richard Ricachenevsky GURSKI, Carlos Cauduro SCHIRMER, Leandro Totti CAVAZZOLA, Ricardo Vitiello SCHRAMM, André Ricardo Pereira ROSA, Cleber Dario Pinto KRUEL

**Affiliations:** 1 Postgraduate Program in Surgical Sciences, Federal University of Rio Grande do Sul, Porto Alegre, RS, Brazil; 2 Department of Digestive Surgery, Federal University of Rio Grande do Sul, Porto Alegre, RS, Brazil; 3 Department of General Surgery, Federal University of Rio Grande do Sul, Porto Alegre, RS, Brazil

**Keywords:** Squamous cell carcinoma of head and neck, Esophagus, Esophagectomy, Neoadjuvante therapy, Carcinoma de células escamosas do esôfago, Esôfago, Esofagectomia, Terapia neoadjuvante

## Abstract

**Background:**

Southern Brazil has one of the highest incidences of esophageal squamous cell carcinoma in the world. Transthoracic esophagectomy allows more complete abdominal and thoracic lymphadenectomy than transhiatal. However, this one is associated with less morbidity.

**Aim:**

To analyze the outcomes and prognostic factors of squamous esophageal cancer treated with transhiatal procedure.

**Methods:**

All patients selected for transhiatal approach were included as a potentially curative treatment and overall survival, operative time, lymph node analysis and use of neoadjuvant therapy were analyzed.

**Results:**

A total of 96 patients were evaluated. The overall 5-year survival was 41.2%. Multivariate analysis showed that operative time and presence of positive lymph nodes were both associated with a worse outcome, while neoadjuvant therapy was associated with better outcome. The negative lymph-node group had a 5-year survival rate of 50.2%.

**Conclusion:**

Transhiatal esophagectomy can be safely used in patients with malnutrition degree that allows the procedure, in those with associated respiratory disorders and in the elderly. It provides considerable long-term survival, especially in the absence of metastases to local lymph nodes. The wider use of neoadjuvant therapy has the potential to further increase long-term survival.

## Graphical abstract

[Fig f1a]

**Figure f1a:**
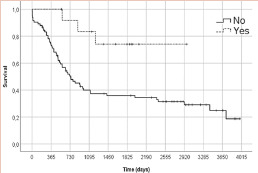
Overall survival in patients who underwent transhiatal esophagectomy submitted or not to neoadjuvant therapy

[Fig f1as]

**Figure f1as:**
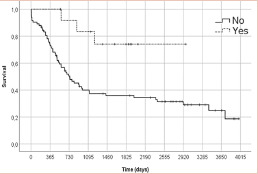
Sobrevida global em pacientes submetidos à esofagectomia transhiatal discriminados por realização de neoadjuvância

## RESUMO

**Racional:**

O sul do Brasil tem uma das maiores incidências de carcinoma epidermoide do esôfago no mundo. A esofagectomia transtorácica permite linfadenectomia abdominal e torácica mais completa do que a transhiatal. No entanto, esta está associado à menor morbidade.

**Objetivo:**

Analisar os desfechos e fatores prognósticos do câncer epidermoide do esôfago que foram tratados com procedimento transhiatal.

**Métodos:**

Foram incluídos todos os pacientes selecionados para abordagem transhiatal como tratamento potencialmente curativo correlacionando sobrevida geral, tempo operatório, análise de linfonodos e uso de terapia neoadjuvante.

**Resultados:**

Foram avaliados 96 pacientes. A sobrevida geral em cinco anos foi de 41,2%. A análise multivariada mostrou que o tempo operatório e a presença de linfonodos positivos foram associados a pior resultado, enquanto a terapia neoadjuvante contribuiu para melhor resultado. O grupo de linfonodos negativos teve taxa de sobrevivência em cinco anos de 50,2%.

**Conclusão:**

A esofagectomia transhiatal pode ser empregada com segurança em pacientes que apresentem desnutrição com grau que permita o procedimento, nos com distúrbios respiratórios associados e nos idosos. Proporciona sobrevida em longo prazo considerável, especialmente na ausência de metástases para linfonodos locais. O uso mais amplo da terapia neoadjuvante tem o potencial de aumentar ainda mais a sobrevida em longo prazo.

## INTRODUCTION

One of the world›s highest incidences of esophageal squamous cell carcinoma (ESCC) is detected in the state of Rio Grande do Sul, Brazil^6^. Most of this population has a low income. Surgical treatment is the standard of care for management with curative intent. Despite advances in surgical techniques and postoperative care in recent years, esophagectomy remains associated with significant morbidity^31^.

Transthoracic esophagectomy (TTE) with two or three operative fields allows for a more complete thoracic lymphadenectomy than transhiatal esophagectomy (THE) does. Although there is no randomized study or systematic review proving oncologic superiority comparing them, the former is considered the standard of care for esophageal cancers. In contrast, in THE patients are spared from thoracotomy and a potential decrease in perioperative morbidity and operative time is expected to occur^10^ ^,^ ^25^ ^,^ ^28^ ^,^ ^31^. Potential disadvantages include the need for blind dissection, especially when lesions are located in upper and middle thoracic esophagus, which may lead to hemorrhage and compromise oncological status^31^. Therefore, it is generally reserved for patients with benign esophageal diseases and in the ones with esophageal cancer whose performance status is lower due to malnutrition or chronic obstructive pulmonary disease.

In contrast to esophageal adenocarcinoma, which usually occurs in well-nourished patients with a history of Barrett’s syndrome secondary to gastroesophageal reflux disease^1^, ESCC usually occurs in malnourished patients secondary to long-term heavy smoking^10^ ^,^ ^25^ ^,^ ^28^ ^,^ ^31^. THE is generally associated with fewer pulmonary complications, and demands less intensive care measures. As the vast majority of our patients are ESCC rather than esophageal adenocarcinoma, we have adopted the policy of performing THE in all patients with malnutrition and respiratory disorders, as well as in older.

There are few prior studies evaluating the outcomes of THE in ESCC and so, the aim of this research was to analyze its results and prognostic factors.

## METHODS

This study was approved by the Research Ethics Committee of Hospital de Clínicas de Porto Alegre (GPPG HCPA 17-0601). Informed consent was waived due to the retrospective, observational design. It included all patients who underwent elective THE as treatment for ESCC at a single center from 2005 to 2017. A gastric tube, by open and laparoscopic technique, was used to reconstruct the gastrointestinal tract - esophagogastrostomy. Data were obtained by reviewing medical records and data from the State Department of Health. Overall survival and specific survival rates were evaluated.

### Operative technique

The operative technique utilized is similar to the described by Orringer^25^. A manual end-to-side esophagogastrostomy with absorbable sutures is performed. The lower edge of the incision remains open for visualization of the viability of the gastric tube and surveillance for anastomotic leaks. A sentinel Penrose drain is placed by counter-incision near the lower edge of the operative wound. Immediate postoperative care is carried out in the intensive care unit. Oral contrast-enhanced examination is performed on the 7^th^ postoperative day to assess the anastomosis.

### Statistical analysis

The primary outcome was postoperative mortality at any time. The secondary was mortality during the first 90 postoperative days. Patients were followed until the end of the study period or until death. The overall survival rate was measured from the date of surgery to the last day of follow-up (in patients who remained alive) or until the date of death. The descriptive variables of interest were age, gender, skin color, smoking status, alcohol intake, hypertension, type 2 diabetes mellitus, chronic obstructive pulmonary disease, lesion size and location, total number of lymph nodes in the pathological specimen, presence or absence of positive lymph nodes, total number of positive lymph nodes, resection margins, neoadjuvant therapy (NAT), adjuvant treatment, intraoperative splenectomy, intraoperative scheduled esophagostomy, operative time, ASA (American Society of Anesthesiologists) classification, length of stay, Clavien-Dindo index, occurrence of anastomotic leak, and other postoperative complications (e.g., pneumonia, cardiac arrhythmia). Survival was analyzed using the Kaplan-Meier method. The log-rank test was used for comparison between different groups. Categorical variables were compared using the chi-square test. Continuous variables were analyzed with the Mann-Whitney U-test or T-test as appropriate. Univariate analysis for each of the two outcomes was performed using the Cox proportional regression method. For the primary and secondary outcomes, the variables considered statistically significant in univariate analysis (p<0.05) were entered into Cox multivariate proportional regression models to identify risk factors independently associated with the two study outcomes. For all analyzes, p-values <0.05 were considered statistically significant. Analyses were performed in SPSS Statistics 18.0 for Windows.

## RESULTS

 The characteristics of the 96 patients included are presented in Table 1. Thirteen (13.5%) underwent NAT. The neoadjuvant protocol included chemotherapy with carboplatin and paclitaxel for five weeks plus radiotherapy (41.4 Gy, divided into 23 fractions). Surgery was planned to occur six to eight weeks after neoadjuvant treatment was finished (Table 1).

**TABLE 1:** Demographic profile of patients undergoing transhiatal esophagectomy for esophageal squamous cell carcinoma (n=96)

Gender	68 men (70.8%)
Age	59.1 (29-84) ± 9.8
Skin color	White: 87 (90.6%) Black: 7 (7.3%) Brown: 2 (2.1%)
ASA	ASA 2: 74 (77.1%) ASA 3: 22 (22.9%)
Neoadjuvant treatment	13 (13.5%)
Adjuvant treatment (1 missing)	8 (8.4%)
Smoking (previous or active) (2 missing)	69 (73.4%)
Alcoholism (previous or active)	33 (34.4%)
Hypertension	26 (27.1%)
Type 2 diabetes mellitus	4 (4.2%)
Chronic obstructive pulmonary disease	9 (9.5%)
Heart disease	7 (7.3%)

Pathological variables are described in Table 2. Postoperative outcomes are seen in Table 3. On univariate analysis, positive margins (HR=2.395, 95%CI=1.337-4.289, p=0.003), positive lymph nodes (HR=2.373, 95%CI=1.420-3.964, p<0.001), size of the tumor (HR=1.014; 95%CI=1.002-1.027, p=0.023), and operative time (HR=1.003, 95%CI=1.001-1.005, p=0.005) were all associated with increased overall mortality. Conversely, NAT (HR=0.245; 95%CI=0.076-0.784, p=0.018) was associated with a 76.5% reduction in overall mortality (Table 4).

**TABLE 2:** Pathological variables of patients undergoing transhiatal esophagectomy for esophageal squamous cell carcinoma (n=96)

Tumor location (1 missing)	3 (upper) (3.2%)	39 (medium) (41.1%)	53 (lower) (55.8%)
Tumor size, cm (2 missing)	2.94 ± 18.4 (0-9.5)		
Median number of lymph nodes	10.8 ± 5.7 (0-27)		
Patients with positive lymph nodes	37 (38.5%)		
Laparoscopy	2 (2%)		
Resection margin (1 missing)	R0 76 (80%)	Not R0 19 (20%)	

**TABLE 3:** Postoperative complications in patients undergoing transhiatal esophagectomy for esophageal squamous cell carcinoma(n=96)

Complication	n (%)		
Pneumonia	42 (43.8%)		
Anastomotic leak	41 (43.2%)		
Cardiac arrhythmia	6 (6.3%)		
Splenectomy	5 (5.2%)		
Need for esophagostomy for delayed cervical anastomosis	11 (11.5%)		
Mediastinitis	5 (5.2%)		
Chilous leak	4 (4.3%)		
Gastric tube necrosis	2 (2.1%)		
Clavien-Dindo >2	44 (45.8)		
	Mean (Std. Dev.)	Median [IQR]	min-max
Operative time (min) (1 missing)	253.7 (101.5)	231 [197; 289]	132-740
Length of stay	24.8 (27.7)	17 [12; 23]	3-201

**TABLE 4:** Univariate analysis of overall survival in patients undergoing transhiatal esophagectomy for esophageal squamous cell carcinoma (n=96)

		Univariate analysis		
		HR	[95%CI]	P
ASA classification	ASA 3	0.669	[0.346; 1.291]	0.231
Heart disease	Yes	0.980	[0.354; 2.712]	0.969
Pneumonia	Yes	0.655	[0.389; 1.104]	0.112
Diabetes mellitus	Yes	0.713	[0.174; 2.931]	0.639
COPD	Yes	1.627	[0.737; 3.593]	0.229
Esophagostomy	Yes	0.994	[0.451; 2.193]	0.989
Splenectomy	Yes	1.268	[0.396; 4.061]	0.689
Alcoholism	Yes	1.312	[0.771; 2.232]	0.317
Anastomotic leak	Yes	0.657	[0.390; 1.108]	0.116
Hypertension	Yes	0.654	[0.352; 1.214]	0.178
Age		0.995	[0.968; 1.024]	0.741
Number of retrieved lymph nodes		0.975	[0.934; 1.018]	0.254
Tumor location	Medium	0.609	[0.182; 2.042]	0.422
	Lower	0.622	[0.190; 2.039]	0.433
Positive circumferential margins	Yes	2.395	[1.337; 4.289]	0.003
Neoadjuvant chemoradiotherapy	Yes	0.245	[0.076; 0.784]	0.018
Presence of positive lymph nodes	Yes	2.373	[1.420; 3.964]	0.001
Gender	Male	0.987	[0.562; 1.733]	0.963
Smoking	Yes	0.691	[0.396; 1.203]	0.191
Tumor size		1.014	[1.002; 1.027]	0.023
Operative time		1.003	[1.001; 1.005]	0.005
Length of stay		0.995	[0.982; 1.008]	0.433
Clavien-Dindo >2	>2	1.465	[0.880; 2.437]	0.142

In the multivariate analysis for overall survival, positive lymph nodes (HR=2.240, 95%CI=1.332-3.769, p=0.002) and prolonged operative time (HR=1.003, 95%CI=1.000-1.005, p=0.019) were the only predictors of increased mortality. Again, NAT was protective, with a nearly 70% reduction in mortality (HR=0.299, 95%CI=0.092-0.970, p=0.044, Table 5). Univariate analysis showed no factors associated with increased 90-day mortality (Table 6).

**TABLE 5:** Multivariate analysis of overall survival in patients undergoing transhiatal esophagectomy for esophageal squamous cell carcinoma (n=96)

		Multivariable analysis		
		HR	[95%CI]	p
Neoadjuvant chemoradiotherapy	Yes	0.299	[0.092; 0.970]	0.044
Presence of positive lymph nodes	Yes	2.240	[1.332; 3.769]	0.002
Operative time		1.003	[1.000; 1.005]	0.019

* Cox regression

**TABLE 6:** Univariate analysis for 90-day survival (n=96)

	Risk Ratio	95%CI	p
ASA classification^#^	0.909	0.183-4.504	0.907
Heart disease	0.625	0.77-5.078	0.660
Pneumonia	41.923	0.105-16691.986	0.221
Diabetes	2.223	0.273-18.076	0.455
COPD	0.301	0.061-1.494	0.142
Esophagostomy	0.890	0.110-7.238	0.914
Splenectomy	0.355	0.044-2.888	0.333
Alcoholism	2.157	0.435-10.690	0.346
Anastomotic leak	53.586	0.173-16607.237	0.174
Hypertension	0.727	0.174-3.042	0.662
Age	1.000	0.928-1.077	0.996
Number of retrieved lymph nodes	0.997	0.882-1.126	0.957
Tumor location			0.489*
Positive circumferential resection margins	0.605	0.117-3.120	0.548
Neoadjuvant therapy	25.316	0.004-152096.807	0.467
Presence of positive lymph nodes	0.628	0.157-2.510	0.510
Gender	1.601	0.800-3.201	0.183
Smoking	1.426	0.288-7.066	0.664
Tumor size	1.026	0.997-1.055	0.80
Operative time	0.999	0.991-1.007	0.764
Clavien-Dindo >2	0.12	0.000-3.713	0.130

**FIGURE 1:**
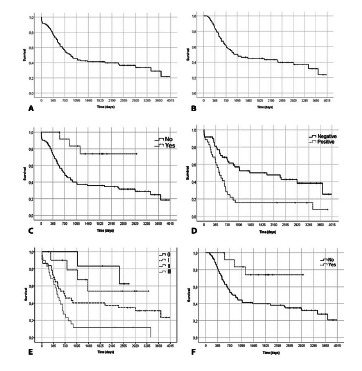
A) Overall survival; B) survival excluding short-term death; C) overall survival in patients submitted or not to neoadjuvant therapy; D) upperfront esophagectomy survival according positive and negative lymph-nodes; E) survival according to TNM stage; F) survival in patients submitted or not to neoadjuvant therapy excluding short-term death.

The 90-day survival was 91.7%, while the 1-year, 3-year, and 5-year survival rates were 76.8%, 46%, and 41.2%, respectively. The median survival was 30.5 months (Figure 1A). Excluding the eight patients who died in the first 90 postoperative days, the 5-year survival rate was 45% (Figure 1B).

Patients who did not receive NAT (n=83) had 1-year, 3-year, and 5-year survival rates of 73.1%, 40%, and 36%, respectively. Conversely, NAT patients (n=13) had 1-year, 3-year, and 5-year survival of 100%, 83.3%, and 74.1% respectively (p=0.01, Figure 1C).

 In the non-NAT group, 35 patients (42%) had positive lymph nodes and 48 (58%) did not. Among those with positive nodes, median survival was 16.6 months and the 5-year survival rate was 15.8%. On the other hand, the negative lymph-node group had a median survival of 65.8 months and a 5-year survival rate of 50.2% (p=0.001, Figure 1D).

 The non-NAT group was separated into stages, according to the 7^th^ edition of the AJCC^29^. Stages II and III comprised 37 (45%) and 29 (35%) patients, respectively. For stage II patients, median survival was 24 months and 5-year survival was 40.4%. For stage III, median survival was 16.6 months and 5-year survival was 11.7% (Figure 1E). Five-year survival excluding 90-day deaths was also analyzed; non-NAT patients achieved a rate of 39.8%, while in the NAT group 74.1% survived (p=0.01, Figure 1F).

## DISCUSSION

 A large number of studies analyzing the results of surgical treatment of ESCC involve cohorts that also comprise esophageal adenocarcinoma^3^ ^,^ ^9^ ^,^ ^15^ ^,^ ^18^ ^,^ ^23^ ^,^ ^33^ ^,^ ^36^ ^,^ ^37^ ^,^ ^38^. Moreover, several studies do not discriminate the results of esophagectomy by tumor type (ESCC vs. esophageal carcinoma). Most papers focusing exclusively on ESCC describe outcomes of TTE rather than THE^21^ ^,^ ^22^ ^,^ ^39^. Moreover, most of these studies include only Asians. Ma et al.^21^ analyzed 695 patients who underwent TTE for ESCC in China. As here, most of the sample was composed of male smokers. In the medical literature, overall survival for TTE in the treatment of ESCC ranges from 17.4% to 41%^11^ ^,^ ^17^ ^,^ ^19^ ^,^ ^40^. 

 Our study analyzes the outcomes of THE employed exclusively in the treatment of ESCC. A total of 96 consecutive cases were included. Although only 16.6% had early tumors, an overall 5-year survival rate of 41.2% was reached. In studies which reported the results of THE for treatment of ESCC^3^ ^,^ ^8^ ^,^ ^11^ ^,^ ^13^ ^,^ ^17^ ^,^ ^19^ ^,^ ^25^, 5-year survival was highly variable (9-48%). Goldminc et al.^12^ published the first prospective randomized study comparing THE vs. TTE. Thirty-two patients underwent THE, achieving a 3-year survival close to 30%, which was similar to the group undergoing TTE. Bogoevski et al.^2^ reported the results of 22 patients with early ESCC (T1a, T1b, and high-grade dysplasia) treated with THE; the 5-year survival rate was 47.6%, similar to that described in our study. Therefore, the outcomes achieved with THE for the treatment of ESCC in our study are comparable to the best previous published results. 

 As expected and reported in the literature, lymph node involvement was associated with worse prognosis in our sample^3^ ^,^ ^19^ ^,^ ^20^ ^,^ ^32^. Multivariable analysis revealed a 2.2-fold increase in overall mortality with metastasis to local lymph nodes. Conversely, circumferential resection margin had no statistically significant risk relationship. This may have been due to the small number of analyzed patients, possibly resulting in type II error.

In the present study, the 5-year survival among lymph node-negative was 53.8%, whereas the lymph node-positive was 20.7%. Evaluating the outcomes in this same objective, Yekebas et al. reported a 5-year survival close to 50%^40^. Lieberman et al.^20^ evaluated 258 patients with esophageal and esophagogastric junction neoplasia who underwent curative esophagogastrectomy (ESCC n=124) and observed that T-stage, N-stage, and number of affected lymph nodes were independent predictors of overall survival, while histological type was not significant. In a recent review, Cho^5^ evaluated the performance of endoscopic ultrasound in the evaluation of lymph node involvement by esophageal cancer and reported up to 99% accuracy of preoperative endoscopic ultrasound with fine needle aspiration in evaluation of metastatic involvement of local lymph nodes.

 In this study, the 5-year survival of patients undergoing upfront surgery was 36%. In parallel, the 13 patients who received NAT (the most recent group in our series) had a 5-year survival of 74.1%. Van Hagen et al.^35^ observed that patients with esophageal and esophagogastric junction neoplasia (adenocarcinoma, ESCC, and undifferentiated carcinoma) who underwent NAT had higher R0 resection rates, important complete pathological response rates (49% for ESCC), and longer long-term survival compared to patients undergoing surgical treatment alone. In the ESCC subgroup, 5-year survival in the NAT group reached about 55%, while the group undergoing exclusive surgical treatment had a survival of approximately 35% over the same period.

 The incidence of anastomotic leak in THE is quite variable in the literature. A systematic review^2^ on esophagectomy complications observed that, although anastomotic leak is the most commonly described complication following esophagectomy, more than 22 different definition criteria for it were utilized. Although efforts are underway to universalize the diagnosis of surgical complications related to esophagectomy, most studies do not present homogeneity in their diagnostic criteria^2^ ^,^ ^26^. Nederlof et al.^24^evaluated the incidence of anastomotic leak in 123 patients undergoing esophagectomy for malignant neoplasia. THE and TTE with end-to-end and end-to-side reconstruction techniques were analyzed. In both groups, anastomosis was constructed in the cervical region using a single continuous layer of monofilament suture. The end-to-end technique had a leak ratio of 22%, whereas end-to-side reconstruction 41%. Among 96 THE with manual end-to-side esophagogastrostomy using absorbable sutures, our sample had a 43% incidence of leakage, similar to that of the end-to-side group of the aforementioned study. Two-step anastomosis has been suggested as a potential strategy to minimize this complication and reduce surgical morbidity and mortality^14^.

 Increased operative time was associated with a modest reduction in survival in the present study. Valsangkar et al.^34^ analyzed 1446 cases of THE between 2010 and 2015 and found that longer operative time in THE was related to higher rates of pneumonia, prolonged intubation, unplanned reintubation, longer hospital stay, septic shock, and mortality.

 One limitation of the current study was the absence of a TTE control group. Although some studies have shown long-term survival benefits for TTE over THE in the treatment of ESCC^17^ ^,^ ^19^ ^,^ ^40^, three previous systematic reviews failed to demonstrate differences between the two procedures^4^ ^,^ ^16^ ^,^ ^30^. Favorable results (5-year survival of 40%) were obtained in patients with stage II cancer in the present study. Considering that previous research has revealed no difference in long-term survival between the two esophagectomy techniques, the results reported herein reveal important internal validity^3^ ^,^ ^7^ ^,^ ^11^ ^,^ ^12^. Donohoe et al. ^7^ concluded that THE can be an alternative to TTE, especially for patients with significant comorbidities or for the treatment of early-stage carcinoma. However, the authors did not discriminate the results of THE specifically for ESCC. The retrospective nature of our design may have led to measurement biases; however, although our review was retrospective, the data were collected prospectively, which may have attenuated these potential biases.

## CONCLUSION

Transhiatal esophagectomy can be safely used in patients with malnutrition degree that still allows the procedure, in those with associated respiratory disorders and in the elderly. It provides considerable long-term survival, especially in the absence of metastases to local lymph nodes. The wider use of neoadjuvant therapy has the potential to further increase long-term survival.
